# Identifying integrins secreted in serum: Unveiling their correlation with inflammation and asthma—A preliminary study

**DOI:** 10.1002/clt2.70023

**Published:** 2025-01-30

**Authors:** Olivia Tellez‐Jimenez, Christian Trejo‐Jasso, Patricia Ramos‐Ramirez, Maryana Tinoco‐Cuellar, Diana García‐Trejo, Angélica Flores‐Flores, Rocío Chapela, José Luis Miguel‐Reyes, Blanca Bazán‐Perkins

**Affiliations:** ^1^ Laboratory of Immunopharmacology Instituto Nacional de Enfermedades Respiratorias Ismael Cosío Villegas Mexico City Mexico; ^2^ Clínica de Asma Instituto Nacional de Enfermedades Respiratorias Ismael Cosío Villegas Mexico City Mexico; ^3^ Laboratorio de Microbiología Molecular Facultad de Medicina Veterinaria y Zootecnia UNAM Mexico City Mexico; ^4^ Tecnologico de Monterrey Escuela de Medicina y Ciencias de la Salud Mexico City Mexico

**Keywords:** Integrin, exacerbation, asthma, cytokines, neutrophil

To the Editor,

Integrins are ubiquitous transmembrane glycoprotein receptors involved in bidirectional signaling across the cell membrane. Integrins are composed of a noncovalent complex that contains an α‐subunit and a β‐subunit, with 18 and 8 variations, respectively, forming a total of 24 distinct heterodimer.^1^ The integrin β1 subunit can interact with 12 α subunits, from α1 to α11 and αv. β2 integrins, along with α4β1, are cell adhesion ligands of leukocyte receptors.^2^ In asthma model, various polypeptides containing both cytosolic and extracellular β1 integrin subunits were detected in airway myocytes and connective tissue, suggesting the secretion of the β1 integrin subunit.^3^ In the context of asthma patients, studies have documented the presence of soluble extracellular domain of α1 and α2 integrin subunits in serum.^4^ However, it remains unclear whether cytosolic integrins domains are present in fluids, such as serum, within the context of the asthma.

In patients with asthma (Supporting Information S1: Table S1), the levels of the extracellular domain of α1 and β1 integrin subunits, as well as the intracellular domain of α1 and β2 integrin subunits, were similar to those of healthy subjects (Figure 1). However, in asthma patients, the levels of the extracellular domain of α2 and β2 integrin subunits, as well as the intracellular domain of α2 and β1 integrin, were higher than those observed in healthy subjects (*n* = 23, *p* < 0.0001). This data is consistent with observations in persistent asthma patients, where an increase in serum soluble α2 integrin (extracellular domain) was observed compared to non‐persistent asthma patients, while α1 integrin remained unchanged.^4^ Interestingly, the expression patterns of integrin β1, α1, and α2 subunits in asthma differed from those observed in scleroderma patients (Supporting Information S1: Figure S1). The integrins α1β1 and α2β1 act as receptors for various types of collagens (I, III, IV, and XIII), each with distinct functions.^5^ Additionally, integrin fragments have the ability to form heterodimers while retaining identical antigenic and ligand‐binding properties as the complete transmembrane integrin.^6^ Therefore, the presence of functional soluble integrin heterodimers in serum is feasible.

**FIGURE 1:**
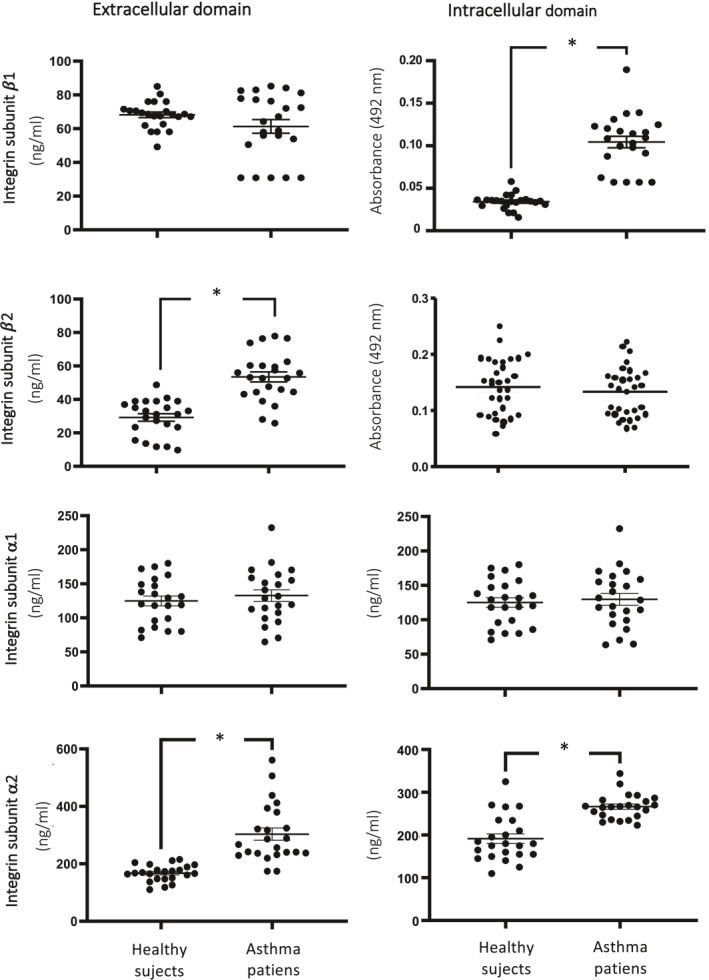
Levels of expression of the intracellular and extracellular domains of integrin α1, α2, β1, and β2 subunits in the serum of patients with asthma. Bars represent means ± standard error, *n* = 23 patients, *p < 0.0001, unpaired Student's *t*‐test.

The changes in the expression of the β1 integrin intracellular domain and both domains of the α2 integrin inversely correlate with FEV1 (Table 1), implying that the severity of asthma may be associated with an upregulation of these integrins. Conversely, the β2 integrin extracellular domain exhibited a direct correlation with FEV1. Supporting this finding, a recent study demonstrated that in asthma patients, this integrin subunit is predominantly released by metalloproteinase‐9 (MMP‐9) during asthma exacerbations, as this is when MMP‐9 levels and activity increase.^7^ Interestingly, despite the lack of correlation between the β2 integrin subunit intracellular domain and FEV1, it does show a direct correlation with the percentage of blood neutrophils, as well as with the levels of IL‐1β, IL‐4, IL‐5, IL‐13, IL‐17, and TNF‐α. Conversely, it inversely correlates with lymphocytes and monocytes. This indicates the potential sensitivity of this subunit to inflammatory cytokines and T2 mediators. Finally, the association of neutrophil levels with the cytosolic domain suggests that these cells could likely be a significant source of cytosolic integrins, and that they might have been released by neutrophil degranulation during serum preparation.

**TABLE 1:** Spearman correlation (*ρ*) between integrin subunit domains in serum with functional and immunological features of asthma patients.

	Healthy controls	Asthma	Integrin subunit β1		Integrin subunit β2		Integrin subunit α1		Integrin subunit α2	
	*n* = 23 (average ± SEM)	*n* = 23 (average ± SEM)	Extracellular domain (ρ)	Intracellular domain (ρ)	Extracellular domain (ρ)	Intracellular domain (ρ)	Extracellular domain (ρ)	Intracellular domain (ρ)	Extracellular domain (ρ)	Intracellular domain (ρ)
FEV1 (% predicted)	92.8 ± 0.7	57.9 ± 3.9#	0.022	−0.765***	0.691***	−0.025	−0.213	−0.009	−0.529**	−0.483*
Lymphocytes (%)	30.2 ± 1.2	25. ± 2.4	0.063	0.227	−0.017	−0.517**	0.06	−0.122	0.159	0.324
Neutrophils (%)	56.6 ± 1.9	64.3 ± 3.5	0.249	0.374	−0.186	0.549**	−0.053	−0.046	0.214	0.137
Eosinophils (%)	4.1 ± 0.4	4.3 ± 0.8	−0.079	0.278	−0.8673	0.2918	−0.153	−0.176	−0.098	0.326
Monocytes (%)	5.9 ± 0.4	5.9 ± 0.6	0.145	−0.055	−0.149	−0.415*	0.117	0.112	0.152	0.304
IL‐1β (pg/mL)	0.58 ± 0.2	0.6 ± 0.2	0.008	−0.017	0.072	0.431*	−0.021	0.103	0.102	−0.010
IL‐4 (pg/mL)	0.36 ± 0.13	0.36 ± 0.1	0.136	−0.019	0.041	0.453*	0.012	0.133	−0.033	−0.194
IL‐5 (pg/mL)	0.43 ± 0.18	0.6 ± 0.3	0.118	0.059	−0.037	0.514*	0.073	0.216	−0.073	0.058
IL‐8 (pg/mL)	3.8 ± 0.9	4.9 ± 1.2	0.143	0.123	0.129	0.154	−0.018	−0.296	0.175	0.058
IL‐13 (pg/mL)	0.64 ± 0.18	0.9 ± 0.2	0.158	0.119	−0.010	0.544**	0.062	0.203	0.022	0.078
IL‐17 (pg/mL)	1.9 ± 0.65	3.2 ± 1.5	0.175	0.052	−0.033	0.409*	0.282	0.265	0.067	−0.082
TNF‐α (pg/mL)	1.5 ± 0.53	2.5 ± 1.2	−0.100	−0.046	−0.044	0.417*	0.082	0.145	0.014	0.048

*Note*: Bold values are significative data.

#*p* < 0.01, unpaired *t*‐test; *p < 0.05; **p < 0.01, paired *t*‐test.

In guinea pigs modeling asthma, the levels of the soluble intracellular domain of β1 and β2 integrin subunits in both control and asthma‐induced models (Supporting Information S1: Figure S2) were comparable in serum and bronchoalveolar lavage (BAL) samples from both young and aged animals. Conversely, the levels of the extracellular domain of these subunits were significantly lower in serum compared to BAL samples (*n* = 6, *p* < 0.05 and 0.01). It appears that the development of acute or chronic lung allergic processes in guinea pigs did not induce changes in serum or LBA β1 and β2 integrin patterns.

In conclusion, our results indicate that integrin secretion occurs in humans and guinea pigs and can be identified in soluble fluids like serum. In humans, the secretion of the β2 integrin subunit may indicate inflammation, while the α2 integrin subunit secretion could signal worsening asthma. The disparities in integrin patterns between asthma and scleroderma underscore the potential of secreted integrin expression as a promising marker for asthma diagnostic.

## AUTHOR CONTRIBUTIONS

Rocío Chapela and Blanca Bazán‐Perkins conceptualized the study design. Olivia Tellez‐Jimenez, Christian Trejo‐Jasso, Patricia Ramos‐Ramirez, Maryana Tinoco‐Cuellar, Diana García‐Trejo, José Luis Miguel‐Reyes, and Angélica Flores‐Flores executed the experiments. Angélica Flores‐Flores and Blanca Bazán‐Perkins performed the interpretation and redaction of the manuscript.

## CONFLICT OF INTEREST STATEMENT

The authors declared that they have no conflict of interest.

## Supporting information

Supporting Information S1

## Data Availability

The study data is available upon reasonable request.
