# Supplementary material for: Identifying integrins secreted in serum: Unveiling their correlation with inflammation and asthma—A preliminary study
Source: Clin Transl Allergy. 2025 Jan 30;15(2):e70023. doi: 10.1002/clt2.70023 (PMC11781838; doi:10.1002/clt2.70023)
Supplement: Supplementary file 1 — Supporting Information S1 [file CLT2-15-e70023-s001.docx]

**Online Supplementary Material**

**Supplementary Methods and Materials**

***Patients with asthma and scleroderma***

All participants in this study were adults, aged 18 and above. In asthma study, each patient from the Asthma Clinic of the Instituto Nacional de Enfermedades Respiratorias had a confirmed diagnosis of asthma, while every healthy volunteer, without any history of lung diseases, familial asthma, allergies, or atopy, had to meet specific inclusion criteria and demonstrate a thorough comprehension of the study's objectives before consenting to participate. Additionally, the exclusion criteria were to decide not to continue participating, to experience an exacerbation of another respiratory disease, or to suffer from any chronic illness or type of fibrosis. Finally, the elimination criteria were: not having a medical diagnosis of asthma, having any respiratory infection, refusing sample collection or being unable to provide it, being pregnant in the case of women, being a smoker, or having any concerns regarding the management of their personal data or information.

The characteristics of asthma patients are summarized in the following supplementary table 1:

|  | **Healthy controls**  (n=23) | **Asthma patients**  (n=23) | ***P* value** |
| --- | --- | --- | --- |
| Gender | Man: 55%  Female: 45% | Man: 50.2%  Female: 49.4% |  |
| Age (years) | 38.6 ± 4.1 | 37.1± 3.5 | *ns* |
| Weight (Kg) | 66 ± 2.0 | 70.8 ± 6.4 | *ns* |
| Body mass index (kg/m^2^) | 25.8 ± 0.9 | 26.7 ± 1.0 | *ns* |

For scleroderma study, 16 female patients meeting the classification criteria for scleroderma were included in the study. They were all with skin involvement and a mean disease duration of over 3 years. None of the patients were currently using corticosteroids or immunosuppressive drugs at the time of the study, nor had they taken them for at least a year. The control group consisted of 16 healthy individuals matched for age, sex, and ethnic background to the scleroderma patients. Furthermore, informed consent was obtained from each patient and healthy volunteer included in this study prior to recruitment. Upon recruitment, a blood sample of 5 cc was collected.

The characteristics of scleroderma patients are summarized in the following supplementary table 2:

|  | **Healthy controls**  (n=16) | **Asthma patients**  (n=16) | ***P* value** |
| --- | --- | --- | --- |
| Gender | Female: 100% | Female: 100% |  |
| Age (years) | 50.4 ± 5.3 | 52.1 ± 4.4 | *ns* |

***Guinea pig asthma model***

We utilized male outbred guinea pigs (0.35 and 0.4 kg, strain HsdPoc:DH) followed protocols approved by the Scientific and Bioethics Committee of the National Institute of Respiratory Diseases. Guinea pigs were sensitized using a combination of ovalbumin (60 μg/ml, Sigma, St Louis, MO, USA) and 1 mg/ml aluminum hydroxide (J.T. Baker, Phillipsburg, NJ, USA), in physiological saline solution (PSS; PISA, Jalisco, Mexico; Fig. Suppl. 1A). After eight days, the sensitization was further enhanced by administering ovalbumin aerosol (5min; 3 mg/ml). Starting from day 15, the guinea pigs were regularly challenged with ovalbumin aerosol for one minute every 10 days at a concentration of 1 mg/ml for the first challenge and 0.5 mg/ml for subsequent challenges. During the third or twelve challenges, the guinea pigs underwent dose–response curve analysis in response to histamine aerosol (ranging from 0.001 to 0.1 mg/ml; Sigma, St. Louis, MO, USA), and tissue acquisition was performed. In contrast, the control group received PSS instead of ovalbumin.

The antigen, histamine, or PSS administration was conducted via acrylic chambers, while pulmonary function was monitored using a whole-body single-chamber plethysmograph designed for freely moving animals (Buxco Electronics Inc., Troy, NY, USA). Data recording was facilitated by the accompanying software (Buxco Bio System XA v1.1, NY, USA), which was utilized for the computation of diverse respiratory parameters, including the bronchoobstructive index (Bi), calculated using the following equation:

Bi = ((Te - Rt)/Rt) (PEP/PIP),

where Te = expiratory time (s), Rt = relaxation time (s), PEP = peak expiratory pressure (cmH_2_O), and PIP = peak inspiratory pressure (cmH_2_O). The aerosols containing ovalbumin, histamine or PSS were generated using a US-1 Bennett nebulizer (flow rate of 2 ml/min; Multistage Liquid Impinger, Burkard Manufacturing Co., Rickmansworth, Hertfordshire, UK), which produced particles of different sizes: < 4 µm (44%), 4–10 µm (38%), and > 10 µm (18%).

***Automated morphometry analysis***

The left caudal lung lobe was isolated and fixed by manual perfusion with a 10% neutral buffered formaldehyde solution through the intra-arterial route until the lung lobe was completely exsanguinated. Lung fragments obtained through sagittal cutting were embedded in paraffin, and 4 µm-thick lung sections were subjected to staining with Masson’s trichrome.

Automated morphometry (Qwin, Leica Microsystems Imaging Solutions, Cambridge, UK) was utilized to determine the surface area (µm^2^) of the subepithelial region (SER). All measurements were performed in six randomly chosen bronchi from each animal. The data were adjusted by the length of the corresponding basement membrane, and their average was considered the result.

***Enzyme-Linked Immunosorbent Assay (ELISA)***

The human and guinea pig fluid samples were centrifuged at 3500 rpm for 10 minutes at 4°C to obtain serum and BAL supernatant. ELISA plates (Costar, USA) were incubated overnight at 4°C with 50 µl of serum from asthmatic, scleroderma patients or healthy volunteers, or with serum or BAL supernatant from guinea pigs, diluted 1:10 in carbonate buffer. A standard curve was generated using known concentrations of each integrin recombinant protein to determine the integrin concentration in ng/ml. The plate volume was decanted, and the wells were washed with a phosphate-buffered saline solution (PBS) (13 mM NaH_2_PO_4_, 88 mM Na_2_HPO_4_, 150 mM NaCl; pH 7.2) containing 0.05% Tween 20 (PBS-T) (Sigma, USA). Non-specific binding sites were blocked with 1% bovine serum albumin (BSA) in PBS-T for 3 hours at 37°C.

Following the incubation, the wells were treated with the following primary antibodies: a monoclonal antibody targeting the extracellular domain of human α1 integrin (mouse-derived, R&D Systems, Minneapolis, USA) at a concentration of 1 µg/ml, and the intracellular α1 integrin domain (mouse-derived, Millipore, Billerica, MA, USA) at a 1:500 dilution; a monoclonal antibody targeting the extracellular domain of human α2 integrin (mouse-derived, Millipore, Billerica, MA, USA) and the intracellular α2 integrin domain (goat-derived, Santa Cruz Biotechnology, Dallas, TX, USA) at a concentration of 1 µg/ml; a polyclonal anti-β1 integrin human antibody against the cytosolic domain (Millipore, Billerica, MA, USA); a monoclonal anti-β1 mouse antibody against the extracellular domain (R&D Systems, Minneapolis, USA) at a dilution of 1:500; a polyclonal anti-β2 human antibody against the β2 integrin subunit cytosolic domain produced in goat (Santa Cruz Biotechnology, Dallas, TX, USA); and a monoclonal anti-β2 human antibody against the extracellular domain produced in mouse (R&D Systems, Minneapolis, MN, USA) at a concentration of 1 µg/ml.

Each antibody was diluted in 1x PBS-Tween 20 containing 0.05% bovine serum albumin fraction V (Sigma, St. Louis, MO, USA). The samples were then incubated overnight at 37°C. After washing the plates, 50 µl of secondary antibody was added. The wells were then incubated for two hours at 37°C with a peroxidase-conjugated secondary antibody derived from horseradish (Sigma, St. Louis, MO, USA). Following this, they underwent a fifteen-minute incubation in a solution containing orthophenylenediamine (Sigma, St. Louis, MO, USA) and 0.2% hydrogen peroxide (Merck KGaA, Darmstadt, Germany). The reaction was terminated by adding 50 μl of 2.5 N sulfuric acid per well. Finally, the absorbance was measured at 492 nm using an automated colorimetric reader (LabSystems, Multiskan model, Tenton, NJ, USA).

The serum levels of various cytokines and growth factors, including granulocyte colony-stimulating factor (G-CSF), granulocyte-macrophage colony-stimulating factor (GM-CSF), interferon-gamma (IFN-γ), and interleukins (IL) such as IL-1β, IL-2, IL-4, IL-5, IL-6, IL-7, IL-8, IL-10, IL-12 (p70), IL-13, IL-17A, macrophage chemoattractant protein (MCP)-1, macrophage inflammatory protein (MIP)-1β, and tumor necrosis factor (TNF)-α, were quantified using the Bio-Plex Pro Human Cytokine 17-plex Assay kit (Catalog # M5000031YV, Bio-Rad, Hercules, CA, USA) on a Bio-Plex MAGPIX System, following the manufacturer’s protocol. Cytokine standards were included on each plate, and both standards and samples were analyzed in duplicate. The prepared bead samples were assessed using the Bio-Plex MAGPIX instrument, which employs dual-laser excitation to read bead-specific and cytokine-specific fluorescence signals. The resulting fluorescent signals were analyzed using Bio-Plex Data Pro software, providing quantitative measurements for each of the 17 cytokines in the samples. Cytokine concentrations (pg/mL) were determined based on the mean fluorescence intensity values of concurrently run standards. Regarding the 17 cytokines measured, only the data showing significant differences with functional and/or serum integrin levels of asthma patients were presented in Table 1.

***Statistical analysis***

All data were subjected to the Shapiro-Wilk normality test. The associations between integrins and patient characteristics were determined by Spearman's correlation coefficient followed of paired t-test. The tests were conducted using the statistical software GraphPad Prism 6.05. To compare multiple groups, repeated-measures ANOVA followed by Bonferroni correction was utilized. Statistical significance was indicated by a two-tailed p value < 0.05. The data in both the text and figures are presented as the mean ± SEM.

***Supplementary Figures***

**Figure S1.** Levels of expression of the intracellular and extracellular domains of integrin α1, α2 and β1 subunits in the serum of patients with scleroderma. Bars represent means ± standard error, n=16 patients, *p<0.001, unpaired Student's t-test.

**Figure S2.** Asthma model in guinea pigs. A) Experimental design. The arrows show when dose-response curves in response to histamine were generated, and bronchoalveolar lavage (BAL) and serum samples were acquired. B) Average maximum (Rmax) broncho-obstructive index (Bi) induced by ovalbumin challenge. C) Determination of the provocative dose 200 (PD_200_) ratio post histamine provocation, calculated as the PD_200_ value observed after antigen challenge divided by the PD_200_ value determined before the challenge. D) Subepithelial region (SER) area. The data were normalized based on the length of the corresponding basement membrane (BM). E) Absorbances of the β1 and β2 integrin subunit in bronchoalveolar lavage (LBA) and serum samples, in control (C) and asthma model (AM) *p < 0.05 and ** p < 0.01 compared with the respective controls, as evaluated by ANOVA followed by Bonferroni correction.
